# Response to Letter Regarding Article: “Manganese-Enhanced Magnetic Resonance Imaging in Takotsubo Syndrome”

**DOI:** 10.1161/CIRCULATIONAHA.123.064230

**Published:** 2023-05-01

**Authors:** T Singh, DE Newby

**Affiliations:** 1 BHF/University Centre for Cardiovascular Science, University of Edinburgh, UK; 2 Edinburgh Heart Centre, Royal Infirmary of Edinburgh, UK; 3 Edinburgh Imaging, University of Edinburgh, UK; 4 Aberdeen Cardiovascular and Diabetes Centre, University of Aberdeen, Aberdeen, UK; 5 Department of Cardiovascular Sciences, University of Leicester and NIHR Leicester Biomedical Research Centre, Glenfield Hospital, UK

**Keywords:** Takotsubo syndrome, Manganese-enhanced magnetic resonance imaging, Myocardial calcium handling

Although the majority of our cohort were rescanned at 3-6 months after the index event, some patients were scanned up to a year later. Reduced myocardial manganese uptake was apparent irrespective of the time period of the second scan [1]. We agree that it will be important to assess whether complete normalisation of manganese uptake occurs beyond one year. We also cannot determine whether altered myocardial calcium handling is a consequence or a cause of the takotsubo syndrome and it is uncertain whether these patients had a ‘healthy’ myocardium to begin with or had a pre-existing cardiomyopathy which made them more susceptible to the acute takotsubo event [2].

Although possible, we believe it is unlikely that absent manganese uptake would occur if we had imaged our patients earlier in the hyperacute phase. Many patients had completely akinetic left ventricular segments but continued to have demonstrable uptake (consistent with viable myocardium albeit with major contractile dysfunction). We believe that absent uptake would be more indicative of a completely infarcted or fibrosed myocardium which is not the case for patients with takotsubo syndrome.

A recent ischaemia reperfusion animal model reported that manganese-enhanced magnetic resonance imaging is an early biomarker of final infarct size after permanent coronary occlusion [3]. Spath et al report that manganese-enhanced magnetic resonance imaging was more sensitive than late-gadolinium enhancement in detecting dysfunctional myocardium [4]. A step-wise reduction was seen in manganese uptake across remote, peri-infarct and infarcted myocardium [4]. After 3 months, myocardial manganese uptake in the peri-infarct region was similar to that of the remote regions, suggesting that the reduced manganese uptake in the peri-infarct area may represent myocardial stunning.

We believe that reduced myocardial manganese uptake reflects abnormal myocardial calcium handling rather than ischemia per se. We excluded patients with obstructive coronary disease although we cannot exclude a contribution of ischemia from microvascular dysfunction or impaired vasoreactivity. Our previous studies have demonstrated reduced manganese uptake patients with acute myocardial infarction, chronic myocardial ischaemia, and those with cardiac risk factors as well as those with dilated cardiomyopathy [5]. However, the patterns of manganese uptake we observed in these populations were very different to that seen in patients with takotsubo syndrome. We therefore feel that microvascular dysfunction alone is unlikely to be the cause of the marked myocardial manganese abnormality.

We demonstrated elevated T1 and T2 values in remote myocardium of patients with takotsubo syndrome reflecting myocardial edema. Due to the nature of quantifying manganese uptake, we captured only a single short-axis slice [1]. As such, we cannot comment on manganese uptake in remote regions, but we agree this is worthy of future study, and that further insights could be gained by studying an animal model of takotsubo syndrome, such as that obtained with cardiac autonomic sympathetic nervous system stimulation.
